# Characterization of the virulence shaping and adaptability in the methicillin-resistant *Staphylococcus aureus* ST9 lineage

**DOI:** 10.1128/msystems.00282-25

**Published:** 2025-06-27

**Authors:** Yiyi Chen, Feiteng Zhu, Yueqin Hong, Yeqiong Liu, Haiping Wang, Shengnan Jiang, Mengzhen Chen, Shujuan Ji, Zhengan Wang, Yunsong Yu, Yan Chen, Lu Sun

**Affiliations:** 1 Center for General Practice Medicine, Department of Infectious Diseases, Zhejiang Provincial People's Hospital (Affiliated People's Hospital), Hangzhou Medical College 117839, Hangzhou, China; 2 Department of Infectious Diseases, Sir Run Run Shaw Hospital, Zhejiang University School of Medicine 56660, Hangzhou, China; 3 Key Laboratory of Microbial Technology and Bioinformatics of Zhejiang Province, Hangzhou, China; 4 Regional Medical Center for National Institute of Respiratory Diseases, Sir Run Run Shaw Hospital, Zhejiang University School of Medicine 56660, Hangzhou, China; 5 Centre of Laboratory Medicine, Zhejiang Provincial People's Hospital, People's Hospital of Hangzhou Medical College 74678, Hangzhou, China; 6 Hangzhou Center for Disease Control and Prevention 117870, Hangzhou, China; Zhejiang University College of Animal Sciences, Hangzhou, Zhejiang, China

**Keywords:** virulence, LA-MRSA, ST9, *agr*, prophage

## Abstract

*Staphylococcus aureus* is a major pathogen responsible for hospital- and community-acquired infections, with the accessory gene regulator (*agr*) system playing a central role in virulence control. The ST9 lineage commonly associated with livestock exhibits multidrug resistance but remains understudied in terms of virulence. In this work, we examined nine clinical ST9 methicillin-resistant *S. aureus* isolates and identified two strains carrying spontaneous frameshift mutations in the *agr* locus, resulting in the loss of RNAIII and α-toxin expression. These *agr*-deficient strains exhibited reduced hemolytic activity, enhanced biofilm formation, and attenuated virulence in a *Galleria mellonella* infection model. These preliminary pieces of evidence suggested a critical role of the *agr* system in ST9 virulence. Comparative genomic analyses revealed distinct pathogenicity islands in ST9, differing from those in other common clinical lineages. Notably, we identified three novel prophage islands (φST9-A, φST9-B, and φST9-C), with φST9-B carrying the recently characterized *tarP* virulence gene, suggesting that recombination events within livestock-associated MRSA could contribute to the genetic diversification of ST9. These findings demonstrate the significant virulence potential of the ST9 lineage and the importance of *agr*-mediated regulation. Moreover, the presence of new prophages highlights the evolutionary adaptability of this lineage. Collectively, our results underscore the clinical threat posed by ST9 MRSA and the necessity for vigilant monitoring to control its spread.

**IMPORTANCE:**

Methicillin-resistant *Staphylococcus aureus* (MRSA) ST9 commonly associated with livestock remains understudied in terms of virulence mechanisms. This study identifies key insights into ST9 MRSA virulence, focusing on the accessory gene regulator (*agr*) system, which regulates virulence in *S. aureus*. We show that *agr*-deficient ST9 strains exhibit altered virulence phenotypes, including reduced hemolysis and increased biofilm formation. Additionally, our genomic analysis reveals novel prophage islands in ST9, highlighting the lineage’s genetic adaptability and potential for increased virulence. These findings emphasize the need for continued surveillance and targeted strategies to control the spread of ST9 MRSA, with important implications for diagnostic and therapeutic approaches.

## INTRODUCTION

Methicillin-resistant *Staphylococcus aureus* (MRSA) is an important pathogen that can colonize in the hospital, community, and livestock environments, causing a wide range of infections, including pneumonia, septicemia, skin and soft tissue infections, osteomyelitis, and endocarditis (1). MRSA harbors a diverse array of virulence factors, including enzymes, surface-associated proteins, pore-forming toxin, superantigens, phenol-soluble modulins, etc. (2). The virulence of a given lineage can be determined either by the specific regulation of an intrinsic factor, such as the accessory gene regulator (*agr*), or the acquisition of virulence factors via mobile genetic elements, such as prophage or pathogenicity islands (3).

Sequence type 9 (ST9) has emerged as the most predominant livestock-associated MRSA (LA-MRSA) in Asia since it was first isolated from pig farms in China (4, 5). Jin et al. reported a highly virulent ST9-SCC*mec*XII MRSA strain causing serious disease in a patient in China (6). Recently, our previous multicenter prospective study uncovered the emergence of ST9 strains in healthcare settings across China and highlighted the multidrug-resistant nature of this lineage (7).

However, the virulence determinants of the ST9 lineage have not been comprehensively characterized. While some studies have attributed ST9 virulence to the presence of immune evasion cluster (IEC) genes, such as *scn*, *chp*, and *sak* (6, 8), the contribution of regulatory systems, such as *agr* and SaeRS, to the virulence of ST9 has not been investigated. In our previous study (7), we performed the genomic analysis of ST9 strains isolated from patients and identified two strains harboring frameshift mutations in the *agr* locus. However, whether these mutations contribute to altered virulence phenotypes remains to be elucidated.

In this study, we investigated the contribution of the *agr* system to the virulence of ST9 lineages by comparing virulence factor expression and virulence phenotypes between *agr* wild-type and -deficient strains. In addition, the pathogenicity island and novel prophages carried by ST9 lineages have been analyzed to gain a more comprehensive understanding of virulence evolution within this lineage.

## MATERIALS AND METHODS

### Bacterial strains and growth conditions

Bacterial strains were cultured in tryptic soy broth (TSB) at 37°C with shaking at 200 rpm. The clinical ST9 MRSA strains used in this study were obtained from a previous multicenter prospective study (9), as also described in our previous research (7). The *agr*-deficient strains were selected for molecular and virulence phenotype experiments, while *agr* wild-type ST9 strains (N28CSA05 and N29CSA11), which differed by the fewest single nucleotide polymorphism from the mutant strains, were used for comparative analyses. The community-associated MRSA ST72 lineage strain HL1 and its *agr*-knockout mutant HL1Δagr (10) were used as controls in virulence experiments.

### Real-time quantitative reverse transcription-PCR (qRT-PCR)

Overnight bacterial cultures were diluted 1:100 into fresh TSB and incubated at 37°C with shaking for 8 h to reach the stationary phase. Total RNA was extracted using the RNeasy Mini Kit (Qiagen), treated with DNase (TURBO DNA-Free Kit, Ambion), and reverse-transcribed into cDNA using the PrimeScript RT Reagent Kit (Takara) following the manufacturer’s instructions. Quantitative PCR was performed using the SYBR Premix Ex Taq PCR Kit (Takara Bio) under PCR conditions of 95°C for 5 min, followed by 45 cycles of 95°C for 5 s, 55°C for 15 s, and 72°C for 15 s using a LightCycler 480 System (Roche). All reactions were conducted in triplicate, with *gyrB* used as the internal control. Each experiment was independently repeated at least three times (11).

### Measurement of α-toxin production

Culture supernatants were collected after 8 h of bacterial growth. Equal volumes of supernatants were loaded onto 15% SDS polyacrylamide gels and electrophoresed at 160 V for 1 h. Proteins were transferred to nitrocellulose membranes using an iBlot Western Blot System (Bio-Rad, America). The membranes were blocked with 5% non-fat dry milk for 1 h at room temperature, followed by overnight incubation with anti-staphylococcal α-toxin mouse serum (1:5,000 dilution; Aladdin) in blocking buffer. After washing three times with washing buffer (Tris-buffered saline, 0.1% Tween-20, pH 7.4), membranes were incubated with horseradish peroxidase-conjugated goat anti-mouse IgG secondary antibody (1:5,000 dilution; Jackson Research) in blocking buffer in the dark for 2 h. Protein bands were visualized using enhanced chemiluminescence detection reagent (BeyoECL Plus, Beyotime, China) according to the manufacturer’s instructions and scanned using a Typhoon Trio+ Variable Mode Imager (GE Healthcare) (10).

### Fitness evaluation

Fitness was assessed based on growth curves and maximum optical density (max OD) values (12). Three independent cultures per strain were grown overnight, diluted 1:100 in TSB, and added in triplicate to a flat-bottom honeycomb 100-well plate. The plates were incubated at 37°C with continuous agitation, and OD_600_ readings were measured every 5 min for 24 h using a Bioscreen C Analyzer (Oy Growth Curves AB Ltd.). Blank wells containing only medium were used to adjust for any changes in OD_600_ unrelated to microbial growth. The max OD was recorded as the OD_600_ value at 24 h. Growth curves were constructed using GraphPad Prism 9.

### Erythrocyte lysis assay

The hemolytic activity of all tested strains was evaluated as previously described (13). Culture filtrates were collected after 8 h of bacterial growth and serially diluted (1:2 to 1:16) in Dulbecco’s phosphate-buffered saline (PBS). Diluted filtrates were then incubated with 2% (vol/vol) human red blood cells at 37°C for 1 h. Hemolysis was quantified by measuring the optical density at 540 nm using a microplate reader. All assays were performed in triplicate.

### Quantitative biofilm assay

Biofilm formation on polystyrene was measured using crystal violet staining, as described by Xie et al. (14). Each strain was cultivated overnight at 37°C, and the cultures were adjusted to a turbidity of 0.5 McFarland standards. A total of 20 µL bacterial suspension and 180 µL TSB medium supplemented with 0.5% glucose were added to a 96-well flat-bottomed microtiter plate in triplicate. After 72 h of incubation at 37°C, the wells were gently washed with PBS three times, stained with 0.1% (w/v) crystal violet (CV) for 10 min, and washed with water. Then, the biofilm-associated CV dye was solubilized with 30% glacial acetic acid for 15 min at room temperature. The OD_570_ of each well was measured.

### Survival assay in *Galleria mellonella* larvae

Strains were grown to mid-exponential phase, washed once with sterile PBS, then resuspended in PBS at 2 × 10^8^ CFU/mL. Fully matured last-instar *G. mellonella* larvae (Tianjin huiyude Biotech, Tianjin, China) were weighed (~300 mg), paired, and randomly grouped to ensure consistent quality for each strain across experimental conditions. Each larva was injected in the buttock with 2 × 10^6^ bacterial cells suspended in 10 µL PBS solution and incubated in clean, sterile petri dishes at 37°C. Mortality was recorded over 72 h to calculate survival rates (15).

### Genomic DNA extraction and sequencing

Genomic DNA was extracted using the Gentra Puregene Yeast/Bact. Kit (Qiagen, Valencia, CA) following the manufacturer’s instructions. DNA concentration was quantified using a NanoDrop 2000 spectrophotometer (Thermo Scientific, Waltham, MA), and DNA integrity was confirmed by agarose gel electrophoresis. A minimum of 5 µg of genomic DNA was required for subsequent library preparation. Sequencing libraries were constructed using the SQK-LSK110 Ligation Sequencing Kit in combination with PCR-Free ONT Native Barcode Expansion kits (EXP-NBD104 and EXP-NBD114, Oxford Nanopore Technologies, UK) following the manufacturer’s protocol without additional DNA shearing, thereby enriching for longer reads. Prepared libraries were quantified by Qubit 4.0 Fluorometer (Invitrogen, CA, USA). Sequencing was subsequently performed on the Nanopore MinION sequencing platform (Oxford Nanopore Technologies, UK).

### Bioinformatics analysis

Virulence genes were confirmed using ABRicate software (https://github.com/tseemann/abricate) with the Virulence Factor Database as the reference. The *agr* locus was aligned using ABRicate with a custom reference database constructed from wild-type *agr* sequences. Hybrid genome assemblies were generated using Unicycler (16), combining second-generation data from a previous study (9) with third-generation data obtained in this study. BLAST was used to compare the ST9 genome with those of other clones to identify pathogenicity islands and discover novel prophage, and Easyfig was used for linear comparison. For strains with only second-generation sequencing data, Mauve and ABRicate were used to confirm the presence of virulence islands by checking gene coverage for integrase and primase enzyme genes coverage. The phylogenetic tree of *S. aureus* ST9 strains was generated in our previous research (7), and virulence genes/phage islands annotations were performed using the iTOL (https://itol.embl.de/) based on this phylogenetic framework.

### Statistical analysis

All statistical analyses were performed using GraphPad Prism software (version 9.0.0; GraphPad Software). For comparisons among multiple groups, either one-way analysis of variance or the non-parametric Kruskal-Wallis test was used depending on data distribution and variance homogeneity. A *P*-value of <0.05 was considered statistically significant. Data were presented as mean ± standard deviation (SD) unless otherwise stated.

## RESULTS

### Identification of *agr* mutations in ST9 MRSA isolates

**Fig 1:**
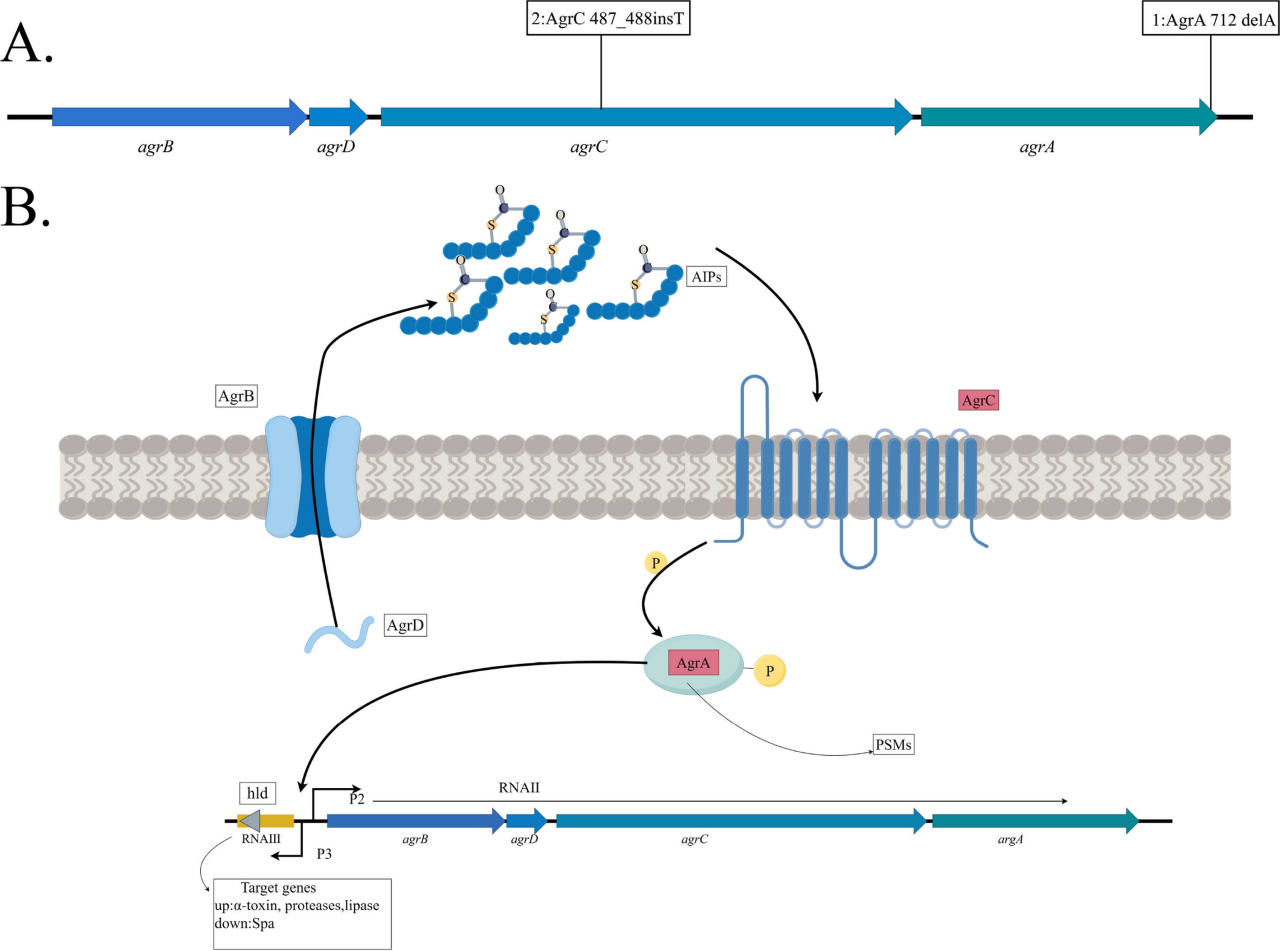
(**A**) Accessory gene regulator (*agr*) system mutations in N08CSA36 and N09HSA31.1 represent the mutation in N08CSA36, and two represent the mutation in N09HSA31. (**B**) Molecular structure, signal synthesis, and transduction cascade of the *agr* quorum sensing system.

Nine ST9 isolates were obtained from our previous study (7), all of which were identified as *agr* type II. Pairwise comparison of the agr gene sequences revealed mutations in two strains: N08CSA36 and N09HSA31. Specifically, strain N08CSA36 harbored a deletion of adenine (A) at position 712 in the *agrA* gene, while strain N09HSA31 exhibited an insertion of thymine (T) between positions 487 and 488 in the *agrC* gene (Fig. 1A). Both mutations resulted in frameshift changes that alter the gene reading frame and prevent gene expression.

To assess the broader prevalence of *agr* mutations in the ST9 lineage, we further analyzed 206 genomes in our phylogenetic analysis (7). Among them, 37 strains exhibited nucleotide sequence variations in the *agr* system (not publication). In addition to N08CSA36, five other strains exhibited frameshift mutations in the *agrA* gene. Similarly, in addition to N09HSA31, three other strains had frameshift mutations in the *agrC* gene. The remaining 27 strains harbored single nucleotide mutations. Most mutations were located in the sensor histidine kinase AgrC and response regulator protein AgrA, suggesting a potential loss of function (Fig. 1B).

### Effect of *agr*-deficient ST9 strains on virulence factor expression

To determine whether these *agr*-deficient ST9 strains effectively deactivate the *agr* regulatory system, we examined the expression of *agr*-regulated genes using *agr* wild-type ST9 strains (N28CSA05 and N29CSA11), ST72 MRSA strain HL1, and its *agr* knockout mutant HL1Δagr as controls.

**Fig 2:**
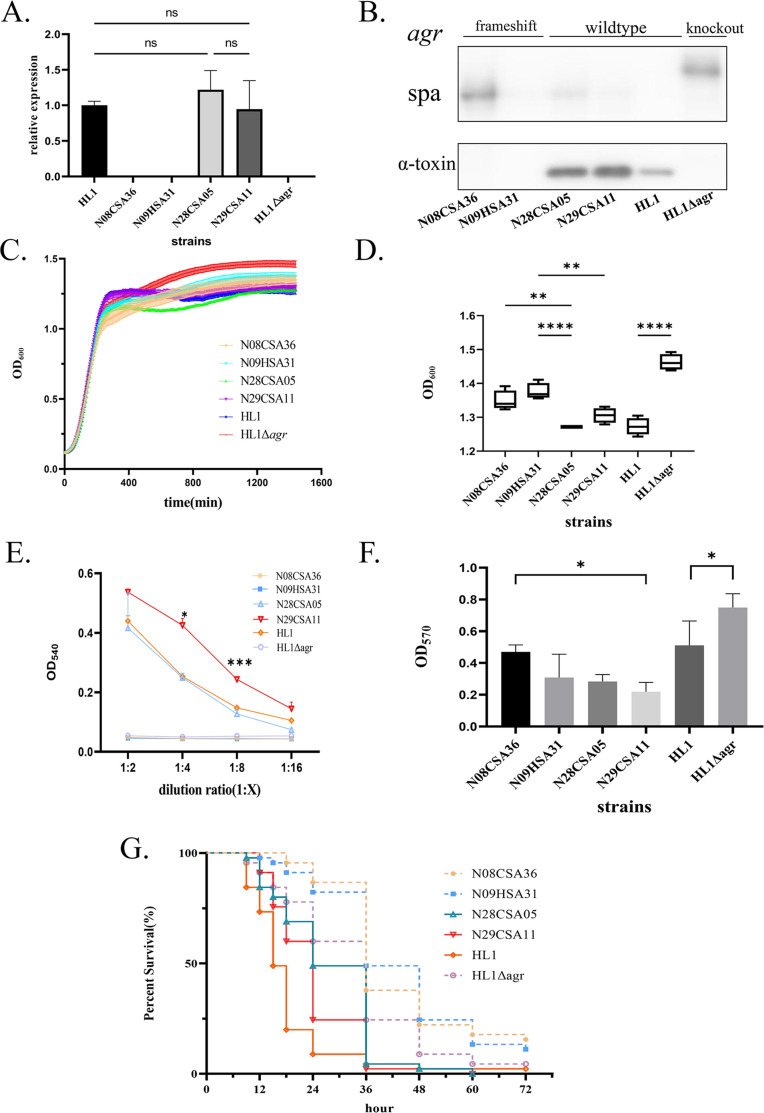
Comparison of virulence phenotypes and genotypes between accessory gene regulator (*agr*)-deficient strains and *agr* wild-type strains. (**A**) Expression of the *agr* system in *agr-*deficient and *agr* wild-type strains. The HL1 strain was used for normalization, with *gyrB* as the internal control gene. The relative expression levels of RNAIII in the *agr* system were shown. ns indicates no significant difference. (**B**) Expression of α-toxin in *agr*-deficient and *agr* wild-type strains. Lanes 1 and 2 represent N08CSA36 and N09HSA31, respectively, both *agr-*deficient strains; lanes 3 and 4 represent N28CSA05 and N29CSA11, respectively, both *agr* wild-type strains; lane 5 represents HL1; and lane 6 represents the HL1 *agr* knockout strain. (**C**) Growth curves of *agr*-deficient and *agr* wild-type strains in the TSB medium. Different colors represent different strains. (**D**) Maximum OD_600_ values. Box plot showing the upper and lower quartiles, median, maximum, and minimum values. Pairwise comparisons of ST9 strains were conducted, and HL1 was compared with its knockout strain. Statistical significance for multiple groups was tested using Šidák’s multiple-comparisons test. **: adjusted *P*-value < 0.01; ****: adjusted *P*-value < 0.0001. (**E**) Biofilm formation ability of *agr-*deficient and *agr* wild-type strains at 72 h. Pairwise comparisons of ST9 strains were conducted, and HL1 was compared with its knockout strain. Statistical significance for multiple groups was tested using Šidák’s multiple-comparisons test. *: adjusted *P*-value < 0.05. (**F**) Erythrocyte lysis assay of *agr-*deficient and *agr* wild-type strains. Dunnett’s multiple-comparisons test was used to compare the hemolytic activity between ST9 *agr* wild-type strains and HL1 at each dilution. *: adjusted *P*-value < 0.05; ***: adjusted *P*-value < 0.001. Significant differences were observed between N29CSA11 and HL1 at dilutions of 1:4 and 1:8. (**G**) Survival assay of *agr*-deficient and *agr* wild-type strains in *Galleria mellonella* larvae. The x-axis represents time, and the *y*-axis represents survival rate. Different colors represent different strains.

The transcription levels of *agr* effectors were tested by RT-PCR in all tested strains. The transcription level of RNAIII was completely abolished in both the *agr*-deficient ST9 strains and the *agr* deletion HL1 strain. Furthermore, the RNAIII expression in *agr* wild-type ST9 strains showed no significant difference from that in HL1 (Fig. 2A).

The expression of α-toxin, regulated by the *agr* system, was detected via western blot analysis in all strains. Both the *agr*-deficient ST9 strains and the *agr*-deletion HL1 strain failed to produce α-toxin but showed expression of surface protein A (Spa), a phenotype consistent with *agr* inactivation. In contrast, the *agr*-functional ST9 strains and the HL1 strain produced α-toxin and exhibited weak or no expression of the Spa protein (Fig. 2B).

### Phenotypic effects of *agr* dysfunction on virulence

All tested strains reached the logarithmic phase at the same time in the TSB medium (Fig. 2C), indicating comparable growth rates between the *agr*-deficient and *agr* wild-type ST9 strains. However, the final OD_600_ of the *agr*-deficient ST9 strains was higher than that of the *agr* wild-type strain (Fig. 2D), which was similar to the behavior observed in the HL1 strain and its *agr* deletion mutant.

Hemolysis (lysis of erythrocytes), a crucial virulence determinant of *S. aureus*, was assessed using an erythrocyte lysis assay. The *agr*-deficient ST9 strains and the *agr* deletion HL1 strain completely lost the ability to lyse red blood cells (Fig. 2E). In contrast, the *agr* wild-type ST9 strains exhibited similar hemolytic ability comparable to HL1. Notably, at 1:4 and 1:8 dilutions, the *agr* wild-type ST9 strain N29CSA11 showed significantly stronger hemolytic activity than HL1.

Biofilm formation in *agr*-deficient and *agr* wild-type ST9 strains was analyzed via crystal violet staining. Strain N08CSA36, which carries a frameshift mutation in *agrA*, exhibited significantly enhanced biofilm formation compared to *agr* wild-type strains, particularly N29CSA11 (Fig. 2F). Similarly, the HL1Δagr mutant formed more robust biofilms than HL1. Although N09HSA31 exhibited a slightly higher biofilm formation than the *agr* wild-type strains, the difference was not statistically significant.

To evaluate the *in vivo* virulence potential of these strains, we compared the survival rates of *G. mellonella* larvae following infection with *agr*-deficient and *agr* wild-type ST9, as well as HL1 and HL1Δagr. As shown in Fig. 2G, the percentage of surviving larvae was significantly higher following infection with *agr-*deficient strains N08CSA36, N09HSA31, and HL1Δagr compared to *agr* wild-type strains N28CSA05, N29CSA11, and HL1 (*P* < 0.0001). The median survival time was 24 h for *agr*-deficient ST9 strains and 36 h for *agr* wild-type ST9 strains. Similarly, the median survival time for HL1 was 15 h, whereas that for HL1Δagr was 36 h, indicating reduced virulence in the absence of a functional *agr* system.

### Virulence factors and pathogenicity islands carried by clinical ST9 MRSA strains

To better understand the virulence potential of the ST9 lineage, we conducted a comparative analysis of virulence genes and pathogenicity islands. As shown in Table 1, ST9 harbored only pathogenicity islands νSa4, νSaα, and νSaβ, while the PVL-containing ΦSa2 found in community-associated MRSA (CA-MRSA) clones ST59, ST1, and ST8 and the immune evasion cluster IEC-containing ΦSa3 found in ST1, ST8, and ST5 were absent in the ST9 clone.

**TABLE 1:** Genomic pathogenic islands and phage carried by clinical ST9 strain^*a*^

Pathogenic island/phage	N29CSA11 (ST9)	SA268 (ST59)	LAC (ST8)	MW2 (ST1)	N315 (ST5)	COL (ST250)
νSa1	−	+	+	−	−	+
νSa2	−	−	−	−	−	−
νSa3	−	−	−	+	−	−
νSa4	+	−	−	+	+	+
νSaα	+	+	+	+	+	+
νSaβ (carrying lukDE)	+	+	+	+	+	+
ΦSa1	−	−	−	−	−	−
ΦSa2 (carrying PVL)	−	+	+	+	−	−
ΦSa3 (carrying IEC)	−	−	+	+	+	−

^*a*^ +, present; −, absent.

Further analysis of the pathogenicity island structure through comparative genomics revealed that the νSaα structure in clinical ST9 strain N29CSA11 contained an insertion of SaPIbov4 (not publication) consistent with the νSaα structure reported in livestock-derived ST9 strains (8). This SaPIbov4 element carried a truncated *vwbp* gene and a homolog of *scn* with 50% sequence identity (not publication), although the function of this gene remains undetermined.

**Fig 3:**
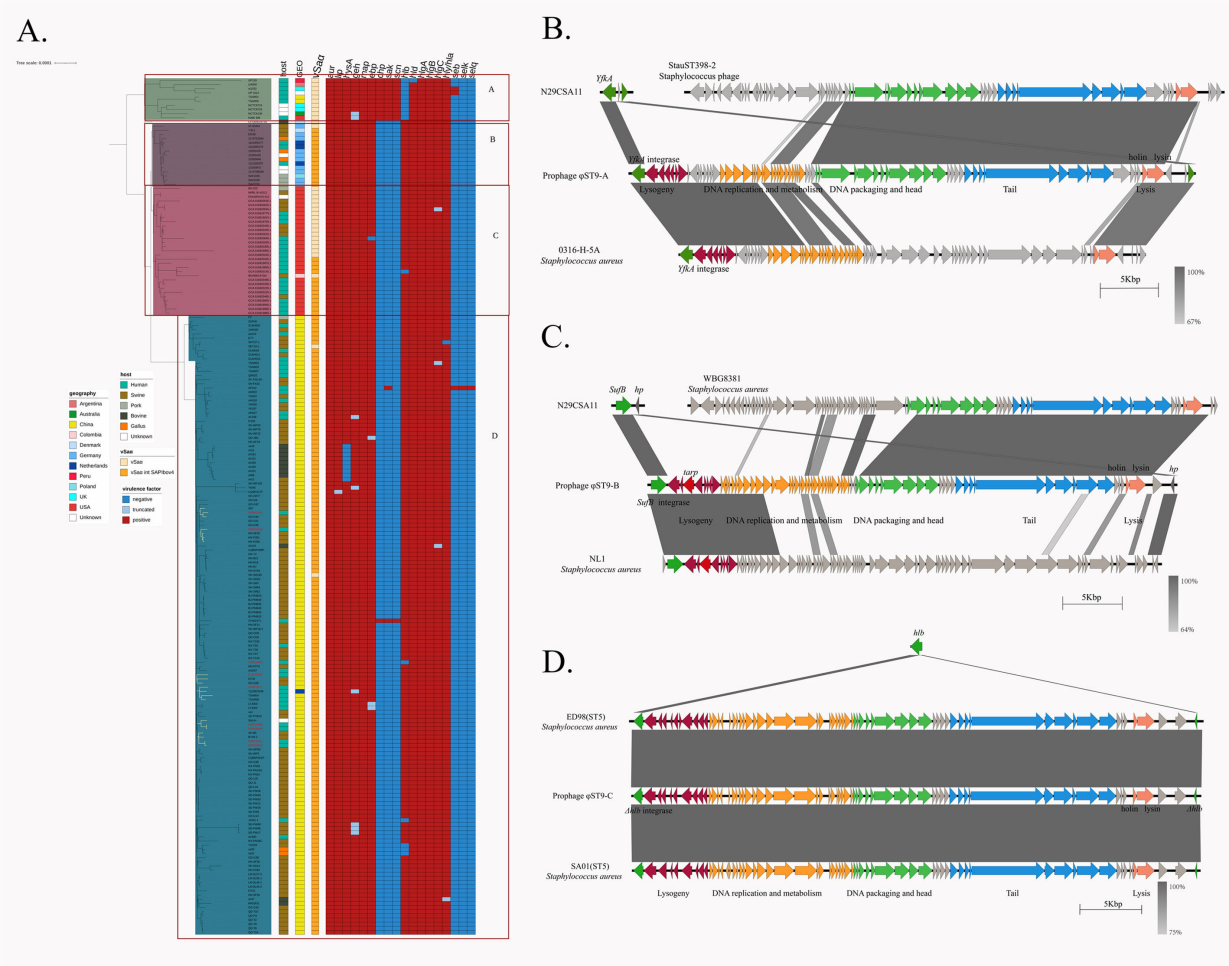
Global distribution of virulence genes in the ST9 lineage and comparison of novel prophage island structures. (**A**) Global distribution of virulence genes in the ST9 lineage. From left to right, the chart represents the host, region, SCC*mec* typing, presence of SaPIbov4 integration in νSaα, and the distribution of virulence genes. Different colors represent specific meanings, as indicated in the legend on the left. (**B**) Structure of prophage φST9-A. (**C**) Structure of prophage φST9-B. (**D**) Structure of prophage φST9-C. Red represents the lysogenic region of the prophage island; green represents the DNA replication and metabolism region; blue represents the tail region of the prophage island; and pink represents the lysis region of the prophage island.

As shown in Fig. 3A, the global virulence gene profiles of ST9 strains were relatively conserved, with most genomes carrying lipase, hyaluronidase, metalloprotease, extracellular adherence protein, and α, β, δ, and γ hemolysins. However, most ST9 strains, except for those in cluster A, lacked the immune evasion cluster, including *scn*, *chp*, and *sak*. Enterotoxins were also rare among ST9 isolates. The νSaα structures in cluster A of the phylogenetic tree and some American strains did not have the SaPIbov4 insertion, whereas most strains isolated from China (cluster D) carried the SaPIbov4 insertion in νSaα, indicating a common origin for this cluster.

### Novel prophage in clinical ST9 MRSA strains

To identify novel prophage islands in the ST9 genome, we selected strains from each clinical subclade according to their distribution in the phylogenetic tree, as constructed in our previous study (7), and performed long-read sequencing to obtain complete genomes. Comparative genomics of clinical strains with complete genome sequences identified prophages φST9-A and φST9-B in N08CSA36 (*agr*-deficient strain) and Prophage φST9-C in N29CSA02.

Prophage φST9-A was inserted between the *yfkA* gene (encoding an uncharacterized radical SAM protein) and a hypothetical gene. This prophage island contained regions for lysogeny, DNA replication, and metabolism, as well as DNA assembly, head, tail, and lytic regions. No known virulence genes were detected. No prophage island with an identical structure to Prophage φST9-A was found in the NCBI database, but it exhibited similarity to a prophage island in the ST398 genome (Fig. 3B).

Prophage φST9-B was inserted between the *sufB* gene (encoding an iron-sulfur cluster assembly protein) and a hypothetical gene. This prophage island also contained regions for lysogeny, DNA replication, and metabolism, along with DNA assembly, head, tail, and lytic regions. In the lysogeny region, we identified the gene *tarP*, a virulence gene discovered in 2018 that has been reported in prophage islands of some ST398 and ST5 strains (17). φST9-B displayed partial structural similarity to prophages from ST398 strain WBG8381 and ST5 strain NL1 (Fig. 3C).

Prophage φST9-C in strain N29CSA02 was inserted into the *hlb* gene (encoding β-hemolysin), the same insertion site of ΦSa3. No virulence genes were identified in this prophage. However, similar structures have been reported in livestock-associated ST5 strains (18) and in ST6324 (a single-locus variant of ST5) isolated from chickens in China (Fig. 3D).

**Fig 4:**
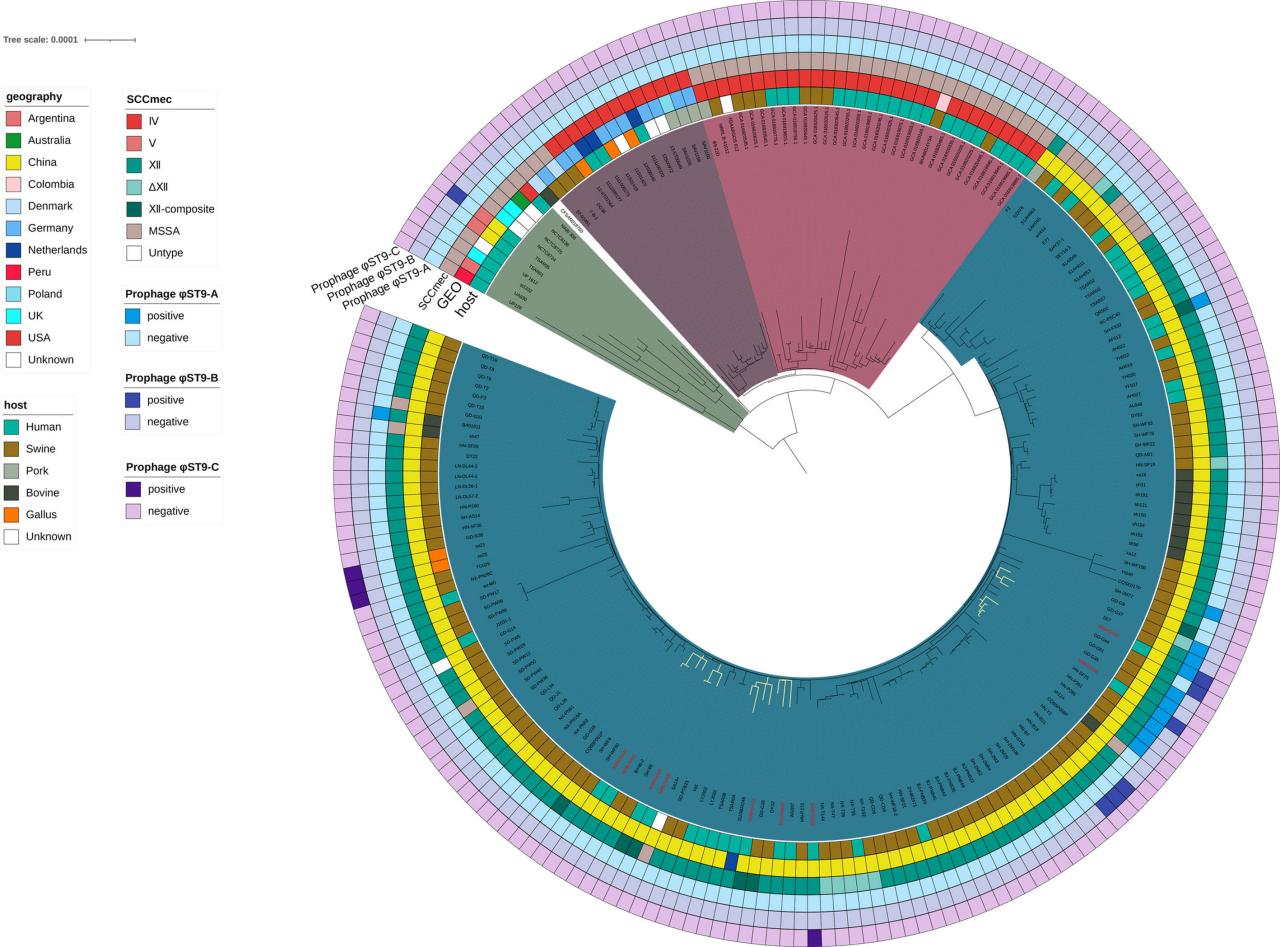
Global prevalence and distribution of the novel prophages in ST9 strains. From inside to outside, each ring represents host, region, SCC*mec* type, and presence of the three prophages. The specific meanings of different colors are provided in the legend on the left.

These novel prophages shared structural similarities with prophages from LA-MRSA lineages, suggesting inter-lineage recombination events in livestock environments. Further analysis of the distribution of these three novel prophage islands in global ST9 strains revealed that these three newly identified prophage islands were exclusively found in a few ST9 strains from China, suggesting that these novel prophage islands are currently locally prevalent (Fig. 4).

## DISCUSSION

The accessory gene regulator (*agr*) system is a well-established quorum-sensing regulator in *S. aureus*, modulating the expression of numerous virulence factors (19). It upregulates several key secreted virulence proteins, including α-toxin, serine proteases, and lipases, while repressing the expression of surface-associated proteins, such as Spa (20). In our study, two ST9 strains harbored frameshift mutations in the *agrA* and *agrC* genes predicted to inactivate the *agr* system. Functional assays confirmed that these mutations led to a loss of RNAIII transcription, decreased α-toxin production, and impaired hemolytic activity. These phenotypic changes are consistent with *agr* inactivation and were validated through RT-PCR, western blotting, and erythrocyte lysis assays. Our results suggest that *agr* dysfunction in the ST9 lineage directly impairs the production of key virulence determinants and alters the virulence profile of these strains. It is important to note, however, that biofilm formation is a complex, multifactorial process influenced by various regulatory pathways. While our results suggest a potential link between *agr* dysfunction and enhanced biofilm formation in certain ST9 strains, *agr* is unlikely to be the sole determinant.

Previous studies have shown that *agr* mutant strains exhibit reduced infectivity in various infection models, including mouse skin infections, acute pneumonia, and rabbit endocarditis (21–23). This underscores the critical role of the *agr* system and its downstream virulence factors in *S. aureus* pathogenicity. However, previous studies on the virulence characteristics of the LA-MRSA ST9 lineage have mostly focused on its pathogenicity islands and virulence genes. For example, Yu et al. have proposed that the host shift of ST9 *S. aureus* from humans to animals involved the loss of IEC genes, such as *scn*, *chp*, and *sak*, which are typically present in human-adapted strains (8). In addition, sporadic cases have reported the presence of IEC-positive ST9 strains (6). However, the role of regulatory systems, such as *agr*, in the virulence of the ST9 lineage has not been elucidated. Our study demonstrates that *agr*-deficient ST9 strains lack α-toxin production, resulting in the loss of hemolytic activity and infection potential. These results indicate that, as an epidemic lineage of LA-MRSA, the virulence regulation mechanism of ST9 is similar to other common clinical lineages, with the *agr* system playing a pivotal role. Furthermore, comparisons with the hypervirulent CA-MRSA clone ST72 (10) revealed that the virulence level of ST9 is comparable to that of ST72, suggesting that ST9 poses a significant clinical threat.

Clinical reports have documented mutations in the *agr* system that result in regulatory dysfunction most commonly occurring in *agrA* or *agrC*, consistent with our findings in the ST9 strains (24). While *agr* dysfunction reduces bacterial invasiveness, clinical studies have indicated that *agr* dysfunction in *S. aureus* is associated with prolonged bacteremia and independently correlates with mortality in critically ill patients (25). Furthermore, *agr* deficiency has been linked to the downregulation of phenol-soluble modulins, contributing to persistent infections and complicating clinical treatment (26). Additionally, research on bacteria isolated from cystic fibrosis patients found that *agr* dysfunction could trigger a proinflammatory response (27). Thus, the *agr* system, as a global regulator in *S. aureus*, can still influence the progression and prognosis of clinical infections, even after inactivation. The emergence of *agr*-deficient clinical ST9 strains highlights the importance of monitoring virulence dynamics during infections caused by this lineage.

Prophages and the pathogenicity islands represent critical mobile genetic elements that encode and disseminate virulence determinants in *S. aureus* (3). The prophage islands in the ST9 genome differ significantly from those in common clinical clones, resulting in a different virulence gene profile. This suggests that ST9 may have distinct origins. Our previous phylogenetic and comparative genomic analyses of antibiotic resistance gene transmission suggested that clinical ST9 strains originated from livestock (7). In this study, we identified several novel prophage islands in clinical ST9 isolates that share structural similarities with those in livestock-associated ST398 and ST5, further supporting the hypothesis that clinical ST9 strains originated from livestock and underwent recombination with other lineages in livestock.

The *tarP* gene first reported in 2018 was initially found in prophage islands of ST398 and ST5 (17). It encodes a glycosyltransferase that replaces the function of TarS, altering the glycosylation site of wall teichoic acid, the major glycoantigen in *S. aureus*, thereby enhancing the bacterium’s ability to evade host defenses (17, 28, 29). It has been reported that *tarP* is also prevalent in other clones, such as CC1 and CC7, in China (30). In this study, we identified *tarP* in a prophage island of ST9 for the first time, suggesting a broader lineage distribution than previously recognized. The potential impact of *tarP* on *S. aureus* virulence and its epidemiological spread warrants further investigation.

This study has several limitations. First, although *G. mellonella* has been demonstrated to serve as an effective infection model in previous studies (31, 32), the absence of additional mammalian models limits the generalizability of our findings. Second, due to the multidrug-resistant nature of ST9 strains, we were unable to perform genetic complementation or knockout experiments to further elucidate the specific roles of the novel pathogenicity islands, the virulence factor *tarP*, and the *agrA/agrC* mutations. Thirdly, since the strains were obtained from a previous multicenter surveillance study, detailed clinical information was not available, preventing us from performing risk factor analyses or correlating *agr* dysfunction with patient outcomes. These limitations highlight the need for more comprehensive models and functional analyses in future research.

In conclusion, this study demonstrates the occurrence of *agr* dysfunction in clinical ST9 strains and establishes that *agr* is crucial for regulating the high virulence potential of the ST9 lineage. Additionally, it reveals that the ST9 lineage has acquired novel pathogenicity islands/virulence factors through genomic exchange in livestock. These findings elucidate the virulence characteristics of the ST9 lineage, underscoring the importance of monitoring its spread in clinical settings.

## Data Availability

The complete genomes of the strains carrying novel prophage islands have been uploaded to the NCBI Genome database with accession numbers CP119571 and CP119341 for chromosomes N08CSA36 and N29CSA02, respectively. The novel prophage island structures have also been deposited in the NCBI GenBank database with accession numbers OQ578993, OQ578994, and OQ578995 for prophages φST9-A, φST9-B, and φST9-C, respectively.
